# Depression and associated factors among prisoners in Bahir Dar Prison, Ethiopia

**DOI:** 10.1186/s12888-019-2071-1

**Published:** 2019-03-11

**Authors:** Fikirte Alemayehu, Fentie Ambaw, Hordofa Gutema

**Affiliations:** 1 Bahir Dar City Health Department, Bahir Dar, Ethiopia; 2 Department of Health Promotion & Behavioral Sciences, School of Public Health, College of Medicine and Health Science, Bahir Dar University, Bahir Dar, Ethiopia; 3 African Mental Health Research Initiative (AMARI) post-doctoral fellow, Addis Ababa University, Addis Ababa, Ethiopia

**Keywords:** Depression, Prisoners, Prison, Ethiopia

## Abstract

**Background:**

Globally there is a rapid increase in prison population, and one out of nine inmates suffers from mental disorders like depression. In Ethiopia, although a mental health strategy is in place, little attention is given to prisoners and studies which focus on depression among prisoners are still scarce. The aim of this study was to assess the prevalence of depression and factors associated with it among prisoners.

**Method:**

Across-sectional study was conducted from October 5 to 28, 2016 in Bahir Dar city. Simple random sampling technique was used to select 402 prisoners. Depression was measured using Patient Health Questionnaire, nine item version (PHQ-9) at a cut point of five. Data on socio-demographic characteristics, behavioral factors, perceived general health, and prison situation variables were collected using structured questionnaire. The data were collected by trained interviewers. SPSS version 20 was used to analyze the data. Binary logistic regression was used to identify predictors of depression.

**Result:**

The prevalence of depression was 45.5% (95%CI: 40.5–50.5%). In the final model, having children [Adjusted Odds Ratio (AOR) = 2.48; 95%CI: 1.60–3.83], health satisfaction rated as moderate [AOR = 3.20; 95%CI: 1.12–9.00] or dissatisfied [AOR = 1.63; 95%CI: 1.02–2.62] compared to satisfied, being sentenced for more than 5 years [AOR = 2.31; 95%CI: 1.01–5.25] or 1–5 years [AOR = 3.04; 95%CI: 1.2–7.71] were positively associated with depression.

**Conclusion:**

High prevalence of depression was found among prisoners. Those with poor general health, long years of imprisonment, and concerns of children were the most vulnerable. Strengthening mental health services of prisons is critically required.

## Background

Depression is a mental disorder characterized by sadness, loss of interest or pleasure, feelings of guilt or low self-worth, disturbed sleep and appetite, feelings of tiredness, lowering of mood and poor concentration. Depending on the number and severity of these symptoms, depression is classified as mild, moderate and severe [1]. Severe depression can lead to suicide, which is the cause for death of over 800,000 people every year worldwide. Globally, around 300 million people are suffering from depression [2].

If the currently existing trends for demographic and epidemiological transition continue worldwide, by the year 2020 the burden of depression will become the second leading cause of disability by increasing to 5.7% out of total disease burden, and it will become the highest cause of burden of a disease in developed countries. Depression is currently the leading disease burden among all mental and physical illnesses, accounting for 10% of total years lived with disability (YLD) in Low- and Middle-Income Countries (LMICs) [1].

Globally, more than 10.35 million people are held in prison. Since the year 2000, the number of individuals imprisoned has increased by about 30% which is higher than the world general population that has grown by 20% during the same period. The prison population rate has increased from 136 to 144 per 100,000 of world population, with big difference among the regions. In the last 15 years, prison population has risen in Latin America with higher (150%) increment in Brazil [3]. The prison population in the USA has decreased until the year 2012, but the figure has started rising again in 2013 [4]. Although it varies among the countries, the prison population has also risen in Asia, Europe, Oceania and Gulf region. In Africa, a large (76%) rise has been observed in Algeria between 2001 and 2003. While the South Africa’s prison population rate has decreased from 394 to 328 per 100, 000 of total country population between 2000 and 2010, Ethiopia’s prison population rate has increased from 94 to 124 per 100,000 of total population between 2000 and 2011 [3, 5].

According to the World Health Organization (WHO), one out of nine prisoners worldwide experience a mental disorder; the majority of them suffer from depression and anxiety. The high rate of mental disorders in this population is due to factors such as overcrowding, violence, enforced isolation, lack of privacy and meaningful activity, isolation from social norm, insecurity about future prospect, and inadequate mental health services in prisons [6–8].

A number of researches conducted in various countries have shown the higher prevalence of depression among prisoners compared to the general population. Prisoners who develop depression are often reported to be at risk of self-harm, developing chronic health conditions and infectious diseases, and having decreased quality of life prior to prison entry [9–13].

In Ethiopia, mental illness is the leading non-communicable disease and depression is among the top-ten high burden diseases. The national prevalence of depression is reported to be 5.0% in the general population. The general public is not well aware of the disease, and this is making health seeking for it low in the country. The Ethiopian Federal Ministry of Health (EFMOH) has developed National Mental Health Strategy based on WHO recommendation by considering prisoners and other vulnerable populations as the groups who need special mental health services [14]. However, only one hospital is providing mental health services for prisoners who are in need and the accurate number of prisoners with mental health disorders is not known. Although the national mental health strategy has given priority to mental health research among prisoners considering the implementation and evaluation of mental health interventions, studies which focus on mental health disorders (including depression) in this population are still scarce in the country. The current study was intended to calculate the prevalence of depression among prisoners and to identify factors associated with depression in this population in Bahir Dar city.

## Methods

### Study design and population

An institution based cross-sectional study was conducted from October 5 to 28, 2016 in Bahir Dar Prison. Bahir Dar Prison is found in Bahir Dar city which is located on the northwest of Ethiopia 565 k meters away from Addis Ababa, the capital of Ethiopia. The total population of the city was 221,991, of which 180,174 (81.16%) were urban residents, and the rest of them were living at rural kebeles around the city. There were about 2200 inmates detained in the only prison in the city.

### Participants and sampling

This study included prisoners whose were 18 years and above. Prisoners who were critically ill and unable to communicate were excluded. The sample size of the study was determined using single population proportion formula by assuming 95% level of confidence, 5% margin of error, and a 50% proportion of depression. By adding 10% for possible non-response rate, the total sample size was 422. Simple random sampling technique was used to select participants. Sampling frame was prepared by obtaining updated list of the all the prisoners from Bahir Dar Prison Office Participants were selected randomly using computer random number generator.

### Data collection procedure and data quality control

Data were collected using interviewer administered structured questionnaire. The English version questionnaire was translated to Amharic and back- translated to English to check accuracy of translation. Four data collectors and one supervisor were employed and two-day training was given for the data collectors and the supervisor on the procedures and ethics of data collection. The questionnaire was pre-tested on 21 participants (5% of the sample size) in another prison in the region, outside Bahir Dar City, before the actual data collection and modification was made based on the finding. The internal consistency of depression assessment tool was checked using Cronbach’s alpha; the tool had an alpha value of 0.78 showing acceptable level of internal consistency.

### Instrument

The questionnaire was comprised of socio demographic characteristics, behavioral factors, health status, and prison situation variables. Patient Health Questioner, nine item version (PHQ-9) [15] was used to assess depression.. Each depressive symptom on PHQ-9 was rated on a scale ranging from zero (not at all) to three (nearly every day). Depression total scores were computed for every one of the participants by adding scores of all the nine items of the scale. A participant was considered to be in the state of depression if he/she scored five and above [16–18]. PHQ-9 had been translated and validated in Ethiopia previously [18]. Social support was measured using Oslo Social Support scale [19]. The scale has been chosen for its extensive use in Ethiopia previously (e.g. [20, 21]), clarity of items, and brevity of the scale.

### Data management and analysis

The data were coded and entered into EPI-Info version-7, and exported to SPSS version 20 for cleaning and further analysis. Descriptive statistical analysis was done to summarize the data. Binary logistic regression was used to analyze the association between the dependent variable (depression) and the independent variables (factors). For all statistical significance tests, the cut- off value was *p*-value < 0.05 with 95% confidence interval.

## Result

### Socio demographic characteristics

Four hundred and two prisoners participated in this study making the response rate 95.3%. More than three fourth (76.6%) of the participants were in the age group of 18–34 years, and the large majority of the participants (98%) were men. Two hundred twenty two (56.7%) of the prisoners were married, and 116 (28.9%) of the participants had never attended school. Ninety eight percent of the participants were Orthodox Christian. Almost half (49.8%) of the prisoners had a child **(**Table 1).

**Table 1:** Socio demographic characteristics of prisoners imprisoned in Bahir Dar prison 2016. *N* = 402

Variable	Frequency	Percent
Age (in years)		
18–34	308	76.6
35–49	62	15.4
> 50	32	8
Sex		
Male	394	98
Female	8	2
Marital status		
Single	172	42.8
Married	228	56.7
Divorced	2	0.5
Religion		
Orthodox	394	98
Protestant	5	1.2
Muslim	3	0.8
Educational status		
Illiterate	116	28.9
Read and Write	55	13.7
Grades1–4	18	4.5
Grades5–8	76	18.9
Grades9–12	97	24
College and above	40	10
Have children		
Yes	200	49.8%
No	202	50.2%

### Health status, imprisonment situation and behavior of the prisoners

One hundred eighty-three (45.5%) [95%CI: 40.5–50.5%] of participant prisoners had depression. Only slightly above half (58%) of the prisoners had satisfactory self-rated general health. Twenty-five (6.2%) of the participants were living with chronic diseases like diabetes mellitus and hypertension. Regarding the duration of imprisonment, 187 (46.5%) of them were imprisoned for 1–5 years, and 177 (44%) of them were imprisoned for less than one year. Two hundred twenty two (55.2%) of the prisoners were sentenced to more than five years. One hundred sixty six (41.3%) of the participant prisoners were convicted of committing murder.

Almost three-fourth (74.4%) of the participants had the habit of attending religious places for three or more days in a week. Regarding the social support, 181(45%) of the prisoners had moderate perceived social support. The majority (78.9%) of the participants were alcohol users, and 65 (16.2%) of the participants were cigarette smokers **(**Table 2).

**Table 2:** Health status, imprisonment situation and behavior of the prisoners imprisoned in Bahir Dar Prison 2016. *N* = 402

Variable	Frequency	Percent
Presence of Depression		
Yes	183	45.5
No	219	54.6
Satisfaction with general health		
Satisfied	233	58
Moderately satisfied	21	5.2
Dissatisfied	148	36.8
Living with chronic illness		
Yes	25	6.2
No	377	93.8
Duration in the prison (in years)		
< 1	177	44
1–5	187	46.5
> 5	38	9.5
Total sentence (in years)		
< 1	51	12.7
1–5	129	32.1
> 5	222	55.2
Type of crime		
Murder	166	41.3
Theft	139	34.5
Physical harm	72	18
Other	25	6.2
Habit of attending religious place		
Not at all	30	7.5
Sometimes in a week	73	18.1
> 3 days in a week	299	74.4
Perceived social support		
Poor	114	28.4
Moderate	181	45
High	107	26.6
Alcohol use		
Yes	317	78.9
No	85	21.1
Smoking cigarette		
Yes	65	16.2
No	337	83.8

### Factors associated with depression

Age, having children, marital status, satisfaction with general health and total year of sentence had significant association with depression during the unadjusted bivariate analysis; however, only having children, satisfaction with one’s own general health and total year of sentence were found to have a statistically significant association with depression in the final logistic regression model after adjusting for confounder variables. Alcohol use, tobacco use and perceived social support didn’t show significant association with depression in the current study.

Prisoners who had children experienced depression 2.48 times more than those who did not have children [AOR = 2.48; 95%CI: 1.60–3.83]. Prisoners who were moderately satisfied or dissatisfied of their general health experienced depression 3.2 and 1.63 times more than those who were satisfied with their general health [AOR = 3.20; 95%CI: 1.12–9.00] and [AOR = 1.63; 95%CI: 1.02–2.62] respectively**.** Inmates who were sentenced to more than 5 and 1–5 years of imprisonment experienced depression 3.0 and 2.3 times more than those who were sentenced to less than one year of imprisonment [AOR = 3.04; 95%CI: 1.2–7.71] and [AOR = 2.31; 95%CI: 1.01–5.25] respectively (Table 3).

**Table 3:** Factors associated with depression among prisoners imprisoned in Bahir Dar prison, 2016. N = 402

Variables	Depression		Crude OR (95% CI)	AOR (95%CI)
	No	Yes		
Age (in year)				
18–34	183	125	1	1
35–49	25	37	2.17 (1.24–3.78) *	1.84 (0.87–3.86)
> =50	11	21	2.8 (1.30–6.00) *	2.28 (0.86–6.04)
Marital status				
Single	109	63	1	1
Ever married	110	120	1.89 (1.26–2.83) *	1.65 (0.81–3.34)
Having children				
No	128	74	1	1
Yes	91	109	2.07 (1.39–3.09) *	2.48 (1.60–3.83) *
Self-rated general health				
Satisfied	142	91	1	1
Moderate satisfaction	8	13	2.54 (1.01–6.34) *	3.20 (1.12–9.00) *
Dissatisfied	69	79	1.79 (1.18–2.71) *	1.63 (1.02–2.62) *
Total sentence (in year)				
< 1	39	12	1	1
1–5	74	55	2.42 (1.16–5.04) *	2.31 (1.01–5.25) *
> 5	106	116	3.56 (1.77–7.15) *	3.04 (1.2–7.71) *

*= significant at *P* value < 0.05; *AOR* Adjusted odds ratio

## Discussion

The current study demonstrated that the prevalence of depression among prisoners in Bahir Dar, northwest Ethiopia to be 45.5%. This figure is massively higher than the national estimate of depression in the general population in Ethiopia which is 5% [14]; this prevalence is also higher than findings of a population-based study conducted in southwest of the country (15%) [22]. Higher prevalence of depression in prison population compared to the general population had been consistently reported from studies done in Nigeria [9, 19],, Brazil [20], Pakistan [11] and Iran [9–11] with some variations in magnitude among the studies. Such variations in the prevalence of depression among the studies might be due to differences in the tools, cut off value used to measure it, variations in prison environments, sample size, and variations in the overall characteristics of the study settings that could be related to the prevalence of depression in one way or another. The possible explanations for the high prevalence of depression among this population could be the higher vulnerability of people with depression for imprisonment or the higher incidence as well as chronicity of depression in prison settings or the combination of these mechanisms. Studies that assess depression states of inmates at the time of imprisonment and following for sometime are required to get some insight about the relative contribution of these mechanisms.

The current study also demonstrated that prisoners who had children experienced depression more than those who did not have children. A study conducted among women incarcerated in the Peshawar Central Prison, Pakistan had reported similar findings [11]. As reported by Munoz-Laboy et al. and Campos et al., lack of close relationship with children might be the reason for high occurrence of depression among inmates [19, 22]. Offenders who had a responsibility of taking care of their children and becoming source of their family income prior to their imprisonment might be in undue stress following the decrease in the income of their family; they might also feel guilty of putting their children under stigma in the community due to their crime. These factors might have increased the emotional distress among the inmates who had children.

The association between self-rated general health and occurrence of depression was also revealed in our study. Prisoners who were only moderately satisfied or dissatisfied of their general health experienced depression more than those who were satisfied with their general health. This finding is in agreement with studies conducted in Nepal [23], Norway [24], and Australia [13]. People with depression may make a negative evaluation of their overall health or people with poor overall health may develop depressive symptoms or get their depressive symptoms persistent more than their counter parts.

Contrary to the findings of Murdoch and colleagues [25], length of time to which the prisoners were sentenced was positively associated with depression in our study. This may be related to the persistently stressful prison environment with inadequate mental health support depleting the coping capacity of inmates [26–28].

One of the limitations of this study was the use of a screening tool to measure depression in which over estimation is likely. However, the scale was previously validated in Ethiopia and was known to have acceptable sensitivity and specificity at a cutoff point we have applied [18]. The other limitation of the study was the utilization of a cross-sectional design which failed to disentangle whether depression was leading to imprisonment or imprisonment was leading to depression. Since the data was collected using interviewer administered questionnaire, this study was also prone to social desirability bias.

### Implications of the findings

Because prisons in a country tend to be similar in terms of facilities, administration and life of prisoners, this study is likely to show the burden of depression in Ethiopian prisons. Repeated systematic reviews have confirmed the high prevalence of depression [29, 30]. Now it is time to advocate for mental health services in prisons. Punishment and correction should not be in the mind of mental health care providers in prisons. An inmate with depressive symptoms may face stigma and discrimination; in some cases suicide may be the end result. On the other hand, prisons benefit from strong mental health services in various ways. For example, a prisoner with untreated depression could impose additional task for prison employees and even to prison mates; therefore, strengthening prison mental health makes prisons appropriate correction institutions where human right violations are absent. Strengthening mental health services in prisons improves the probability that upon leaving prison they will be able to adjust to community life, which may, in turn, reduce the likelihood that they will return to prison [31].

## Conclusion

In this study, the magnitude of depression among inmates was high. Prisoners who had children, dissatisfied with their general health, and those who were sentenced to more than a year had depression more than their counterparts. Hence**,** the implementation of programs that strengthen effective coping strategies, improve close relationship of prisoners with their families, and promoting satisfaction with their own general health are likely to increase the psychological well-being of inmates. Prisoners sentenced to longer years of imprisonment require greater attention. Further studies are required to sort out whether imprisonment per se leads to depression or not.

## Abbreviations

CI: Confidence Interval
EFMOH: Ethiopian Federal Ministry of Health
OR: Odd Ratio
OSS: Oslo Social Support
PHQ: Patient Health Questionnaire
SPSS: Statistical Package for Social Sciences
WHO: World Health Organization
